# Serotonin receptor 5-HT7 modulates inflammatory-associated functions of macrophages

**DOI:** 10.1007/s00018-024-05570-z

**Published:** 2025-01-21

**Authors:** Frauke S. Bahr, Franziska E. Müller, Martina Kasten, Nils Benen, Irina Sieve, Michaela Scherr, Christine S. Falk, Denise Hilfiker-Kleiner, Melanie Ricke-Hoch, Evgeni Ponimaskin

**Affiliations:** 1 Cellular Neurophysiology, Hannover Medical School, Hannover, Germany; 2 Department of Cardiology and Angiology, Hannover Medical School, Hannover, Germany; 3 Centre for Pharmacology and Toxicology, Hannover Medical School, Hannover, Germany; 4 Department of Hematology, Hemostasis, Oncology and Stem Cell Transplantation, Hannover Medical School, Hannover, Germany; 5 Institute of Transplant Immunology, Hannover Medical School, Hannover, Germany; 6 German Center for Infection Research, DZIF, TTU-IICH, Hannover-Braunschweig Site, Hannover, Germany; 7 Medical Faculty of the Philipps-University Marburg, Department of Cardiovascular Complications of Oncologic Therapies, Marburg, Germany

**Keywords:** Macrophages, THP-1 cells, Serotonin, 5-HT7 receptor, Phagocytosis

## Abstract

The hormone and neurotransmitter serotonin regulates numerous physiological functions within the central nervous system and in the periphery upon binding to specific receptors. In the periphery, the serotonin receptor 7 (5-HT7R) is expressed on different immune cells including monocytes and macrophages. To investigate the impact of 5-HT7R-mediated signaling on macrophage properties, we used human THP-1 cells and differentiated them into pro-inflammatory M1- and anti-inflammatory M2-like macrophages. Pharmacological 5-HT7R activation with the specific agonist LP-211 especially modulates morphology of M1-like macrophages by increasing the number of rounded cells. Furthermore, 5-HT7R stimulation results in significantly reduced phagocytic and migratory ability of M1-like macrophages. Noteworthy, LP-211 treatment leads to changes in secretory properties of all macrophage types with the highest effects obtained for M0- and M2c-like macrophages. Finally, the importance of 5-HT7R for regulation of phagocytosis was confirmed in human primary CD14^+^ cells. These results indicate that 5-HT7R activation selectively impairs basic functions of macrophages and might thus be a new access point for the modulation of macrophage responses in the future treatment of inflammatory diseases.

**Supplementary Information:**

The online version contains supplementary material available at 10.1007/s00018-024-05570-z.

## Introduction

Serotonin (5-HT) is a neurotransmitter of the central nervous system known to modulate mood, pain, body homeostasis, and circadian rhythm [1, 2]. However, nearly 95% of the body’s 5-HT is produced and released by enterochromaffin cells in the gut, where it acts locally or it can enter into the blood circulation. In the periphery, 5-HT acts as a hormone to modulate various physiological processes via activation of serotonin receptors belonging to seven major families, including the serotonin receptor 7 (5-HT7R) [3–6]. The 5-HT7R belongs to the family of G protein-coupled receptors and can activate two different heterotrimeric G proteins. The canonical signaling involves activation of the Gs-protein and adenylyl cyclase (AC), leading to increased synthesis of cyclic adenosine monophosphate (cAMP) in the cell [7–9]. In addition, 5-HT7R can activate the G12-protein resulting in activation of small GTPase cell division control protein 42 homolog (Cdc42) leading to modulation of cytoskeleton [10, 11].

The 5-HT7R was shown to be an important regulator of the immune system [2, 12]. Among others, 5HT7R-mediated signaling was identified to be crucial for T lymphocyte activation [13]. Our own data and results from other groups demonstrated that 5-HT7R expressed on dendritic cells regulates their maturation and morphology and could modulate cytokine and chemokine secretion [14–16]. Moreover, human lipopolysaccharide (LPS)-primed monocytes display changes in their secretion profile upon 5-HT7R stimulation [17]. It has also been demonstrated that 5-HT7R- and 5-HT2BR-mediated signaling influence in vitro monocyte-to-macrophage differentiation [18, 19]. In addition, macrophages can alter their gene expression profile via 5-HT7R - protein kinase A (PKA) signaling pathway [20]. However, the detailed role of 5-HT7R-mediated signaling in the regulation of the basic macrophage functions has not yet been investigated.

Macrophages display a high degree of phenotypic and functional heterogeneity. In vitro, two main macrophage subtypes are conventionally discriminated: Classically activated macrophages, which display a pro-inflammatory phenotype (M1-like), and alternatively activated macrophages of various subtypes with anti-inflammatory properties (M2-like) [21–23]. The identification of each subtype can be achieved through the determination of surface marker expression. For instance, M1-like macrophages typically express high levels of CD80 and human leukocyte antigen-DR isotype (HLA-DR), whereas M2a-like macrophages can be identified by their expression of CD206 and M2c-like macrophages by CD163 [24]. One important feature of both types of macrophages is phagocytosis of pathogens and necrotic tissue to clear the area of inflammation. Another important characteristic of macrophages is the initiation of inflammatory responses via secretion of cytokines and chemokines. Depending on their phenotype, distinct pro- or anti-inflammatory environments can thereby be achieved, which attracts various immune cells.

Human THP-1 cells are a broadly used and well-characterized cell culture model for investigation of macrophage functions in vitro. This monocytic, non-adherent cell line was isolated from a 1-year old male with acute monocytic leukemia [25, 26]. Treatment of THP-1 cells with phorbol-12-myristat-13-acetat (PMA) or macrophage colony-stimulating factor (M-CSF) leads to cell adherence and initiates differentiation of monocytes to macrophages [25, 27–31]. The latter can be further differentiated into macrophage subtypes with pro- or anti-inflammatory properties, M1- and M2-like macrophages, respectively. Similarly, CD14^+^ cells isolated from human peripheral blood monocytes (PBMCs) and differentiated into the respective macrophage subtypes represent a suitable model for analysis of macrophage functions [32]. Although such traditional classification (i.e., M1, M2a, M2c), which is based on induction of in vitro polarization, does not accurately describe the full phenotypic heterogeneity of in vivo macrophages, for ease of reading, we will use this classification throughout this work [25].

In this study, we verified expression of 5-HT7R on different subtypes of THP-1-derived and CD14^+^ macrophages and investigated the functional role of this receptor on macrophage properties. Among others, we analyzed whether the activation of the 5-HT7R by the selective agonist LP-211 modulates cell morphology, migration, phagocytosis, and secretory properties.

## Materials and methods

### THP-1 cell culture

THP-1 cells were purchased from ATCC (TIB-202) and cultured in RPMI medium (ThermoFisher, 51800-019) supplemented with 10% FBS superior stabil (Bio&Sell, S 0615) and 1% Penicillin (100 U mL^− 1^)/Streptomycin (100 µg mL^− 1^; Gibco, 15070-063). The differentiation protocol was modified from Surdziel et al. [30]. In brief, THP-1 monocytes were split (day 1) and respective cell numbers were seeded according to the cell culture dish (0.5 × 10^6^ cells mL^− 1^). For differentiation to macrophages, phorbol-12-myristat-13-acetat (PMA; Sigma, P8139-1MG, 20 ng mL^− 1^) solved in dimethyl sulfoxide (DMSO, Sigma Aldrich, 276855-100ML, 100%) was added and renewed after 24 h. On day three, the medium was exchanged and macrophage polarization was initiated. M0 macrophages received plain medium; to obtain M1-like macrophages - interferon gamma (IFN-γ; Peprotech, AF-300-02, 20 ng mL^− 1^) and lipopolysaccharide (LPS; Sigma, L8274, 100 ng mL^− 1^) were added, for M2a-like macrophages the medium was supplemented with interleukin-4 (IL-4; Peprotech, AF-200-04, 20 ng mL^− 1^) and for M2c-like macrophages with IL-10 (Peprotech, AF-200-10, 50 ng mL^− 1^). On day 5, the THP-1 derived macrophages were used for experiments.

### Isolation and differentiation of CD14^+^ cells

Peripheral blood mononuclear cells (PBMCs) from healthy donors were isolated as described in Sieve et al. [32]. In brief, the filters from platelet apheresis received form the blood transfusion service at Hannover Medical School were perfused with 50 mL of PBS (Sigma-Aldrich; P4474) and loaded with 12.5 mL of Biocoll Separating Solution (1.077 g mL^-1^, Biochrom, L6715). Subsequently, the mononuclear cells were pelleted (300 g for 30 min at room temperature (RT)), transferred and resuspended in PBS. Following an additional centrifugation step (300 g for 10 min), the cells were counted. To isolate CD14^+^-cells, MACS^Ⓡ^ technology was used in accordance with the manufacturing protocol using anti-human CD14-MicroBeads (Miltenyi Biotec, 130-050-201). 1 × 10^6^ cells mL^-1^ were seeded into RPMI 1640 medium (Gibco, 21870076) supplemented with 10% FBS (Biochrom, S 0615) and 1 mM L-glutamine (Gibco, 25030081). Basal cells were cultured during the whole experiment in plain medium. To differentiate the cells into M1-like cells, granulocyte macrophage colony-stimulating factor (GM-CSF; Peprotech, 300-03, 50 ng mL^-1^) was added. To gain M2-like macrophages, macrophage colony-stimulating factor (M-CSF; Peprotech, 300 − 25, 50 ng mL^-1^) was supplemented. The medium and stimuli were renewed on day four of the differentiation. On day 2, 4, 7, and 10, the cells were utilized for 5-HT7R mRNA experiments, whereas all other experiments were performed on day 7 after seeding.

### Flow cytometry

THP-1-derived macrophages were washed with prewarmed PBS, incubated with 0.25% Trypsin-EDTA (1X), phenol red (Invitrogen, 25200-56) for 10 min at 37 °C and detached by gentle pipetting. Cells were pelleted (400 g, 5 min, 4 °C) and suspended in 1 mL autoMACS Rinsing Solution (Miltenyi Biotec, 130-091-222). All stainings were performed with 1 × 10^7^ cells and centrifugation steps were performed at 400 g for 5 min at 4 °C. Blocking was done with donkey serum (1:100, Jackson Immunoresearch, 017-000-121) or Human TruStain FcX (1:100, Biolegend, 422301) for 5 min, followed by centrifugation and incubation with antibody mix (PE/Cyanine7-anti-human CD11b (Biolegend, 301321), PerCP/Cyanine5.5-anti-human MERTK (Biolegend, 367621), APC-anti-human HLA-DR (Biolegend, 307610), PE-anti-human CD86 (B7-2; eBioscience, 12-0869-41); BV421-anti-human CD163 (BD, 562643), PE/Dazzle 594-anti-human CD105 (Biolegend, 323223), Alexa Fluor 700-anti-human CD14 (Biolegend, 367113), PE-anti-human CD200R (Biolegend, 329306), FITC-anti-human CD64 (Invitrogen, 11-0649-42) for 20 min at 4 °C, washing, centrifugation and resuspension in autoMACS Rinsing Solution. Stained cells were measured at Sony Spectral cell analyzer SA3800 using FCS Express 6 software for data analysis.

### Western blot

THP-1 cells were differentiated as described above and lysed on day 5 of differentiation. Adherent cells (M0, M1, M2a, and M2c) were washed once with cold PBS before adding RIPA lysis buffer and supplemented with protease inhibitors CLAP and PMSF. Cells were detached using a cell scraper and lysates were transferred into reaction tubes. Undifferentiated THP-1 cells in suspension were centrifuged at 700 rpm for 7 min and washed once with PBS. The cell pellet was resuspended in lysis buffer. All lysates were centrifuged at 15 000 g at 4 °C for 15 min and the supernatant was used for further experiments. Protein concentrations were determined using Pierce BCA Protein Assay Kit (Thermo scientific, 23225) according to the manufactures protocol. Supernatants were mixed with 6x Nick loading buffer with β-mercaptoethanol (Carl Roth, 4227.1, 5%) and loaded on an SDS-gel (12%) run at 140 V. After separation, the proteins were transferred onto nitrocellulose membranes (Cytiva, 10600003) and blocked for 1 h at RT with milk powder (5%) in TBS-T. Membranes were incubated overnight with following primary antibodies: 5-HT7R (1:1000, Abcam, 128892), Gαs (1:500, Abcam, ab101736), Gα12 (1:250, Santa Cruz, sc-515545), Cdc42 (1:500, 610929, BD Biosciences, in SignalBoost™Immunoreaction Enhancer Kit, Merck Millipore, 407207), GAPDH (1:5000, Millipore, MAB374) in milk (5%), if not differently specified. Membranes were washed three times with TBS-T and incubated with corresponding secondary antibodies (all 1:10000 in milk (5%), goat anti-rabbit IgG (H + L) HRP (Thermo Fisher Scientific, 31460), rabbit anti-mouse IgG Fc HRP (Thermo Fisher Scientific, 31455), rabbit anti-goat IgG (H + L) HRP (Thermo Fisher Scientific, 31402)) for 1 h. The western blots were developed using SuperSignal West Femto Maximum Sensitivity Substrate (Thermo Fisher Scientific, 3457734096), SuperSignal West Pico Plus Chemiluminescent Substrate (Thermo Fisher Scientific, 34577), or Immobilon ECL Ultra Western HRP Substrate (Merck, WBULS0100) respectively at Fusion SL Vilber Lourmat (PeqLab). For quantification of the band signals, a custom-written Matlab script was used and normalization was done by the sum of replicates method.

### Quantitative real-time polymerase chain reaction (qRT-PCR)

RNA from THP-1-derived macrophages was isolated using RNeasy Mini Plus Kit (Qiagen, 74134) whereas RNA from CD14^+^ was harvested using TRIzol Reagent (Thermo Fisher Scientific, 15596026). The RNA was transcribed with either SuperScript^®^ III First-Strand Synthesis System (Invitrogen, 18080-051) or LunaScript™ RT SuperMix Kit (NEB, E3010L). For detection of differentiation marker expression an AriaMx Realtime-PCR System (Agilent Technologies, Software Agilent Aria 1.5) with Maxima SYBR Green qPCR Master Mix (2x) (ThermoScientific, K0253) was used. The respective sequences of the utilized primers are listed in Table 1.

**Table 1:** Primer sequences

Gene	Forward	Reverse
5-HT7R	CTTCGTCAAGAAGCTCCGCC	TGTGATCCCAAGGTACCTGTCAA
18 S	AGAACGAAAGTCGGAGGTTCG	GGACATCTAAGGGCATCACAG
CCL5	TCATTGCTACTGCCCTCTGC	TCGGGTGACAAAGACGACTG
CCL22	TTACGTCCGTTACCGTCTGC	CCACGGTCATCAGAGTAGGC
CD163	TTTGTCAACTTGAGTCCCTTCAG	TCCCGCTACACTTGTTTTCAC
CD206	GCCAAATGACGAATTGTGGA	CACGAAGCCATTTGGTAAACG
CD80	TTGGATTGTCATCAGCCCTGC	ATTTTCTCCTTTTGCCAGTAG
Cdc42	GGTGGAGAAGCTGAGGTCAT	CATCGCCCACAACAACACAC
CXCL10	GCACCATGAATCAAACTGCCA	CCTCTGTGTGGTCCATCCTTG
Gα12	GTGAAGATCCTGCTGCTGGG	AACCCTTGAGCCCTTGAGGA
Gαs	AGTAAGACCGAGGACCAGCG	TGCCTTCTCACTGTCTCCATT
IL-1β	AAACCTCTTCGAGGCACAAG	GTTTAGGGCCATCAGCTTCA
IL-6	AATTCGGTACATCCTCGACGG	TTGGAAGGTTCAGGTTGTTTTCT
IL-8	AGAACGAAAGTCGGAGGTTCG	GGACATCTAAGGGCATCACAG
MCP-1	CCTTCATTCCCCAAGGGCTC	GGTTTGCTTGTCCAGGTGGT
MIP-1a	GCTCTCTGCAACCAGTTCTCT	GAAGCTTCTGGACCCCTCAG
TNF-α	CGCCACCACGCTCTTCTG	GCCATTGGCCAGGAGGGC

Expression levels of 5-HT7R signaling pathway components in THP-1-derived macrophages were determined on a StepOne Plus System (Applied Biosystems) with either TaqMan Universal PCR Master Mix (Applied Biosystems, 4324018) or Luna^®^ Universal Probe qPCR Master Mix (M3004L, NEB). The following Taqman Gene Expression Assays (Thermofisher), primers and probes were used: 5HT7R (Hs00909028_g1), Gα12 (Hs00170899_m1), Cdc42 (forward: AGAAAAGTGGGTGCCTGAGAT, reverse: AATTTGAGTCCCAACAAGCAA, probe: GTCACCACTGRCCAAAGACTCCTT), Gαs (Hs00255603_m1), and β-actin (Hs99999903_m1). Relative mRNA expression levels were calculated using the 2^−∆∆CT^ method.

### Immunocytochemistry and microscopy

#### Bright field

Differentiated THP-1 cells were imaged in a 48-well plate using bright field at Zeiss Axio Oberver.Z1/7, with Zen 3.2 Blue software (Carl Zeiss, Jena).

#### Immunofluorescence staining and imaging

THP-1-derived macrophages were grown on glass cover slips and fixed using paraformaldehyde (PFA, Carl Roth, 0335.3, 4%) for 10 min at RT, permeabilized with acetone for 3 min at -20 °C, washed with PBS and unspecific binding sites were blocked with albumin fraction V (Carl Roth, T844.4, 1%) in PBS for 1 h at RT. Afterwards, cells were incubated with anti-5HT7 Receptor/HTR7 (extracellular)-FITC antibody (1:400 in PBS, Alomone Labs, ASR-037-F) or 5HT7 Receptor/HTR7 (extracellular) Blocking Peptide (1:10 ratio to the antibody according to the manufacturers recommendations; Alomone Labs, BLP-SR037) for 1 h at RT. Nuclei were stained using DAPI (1:5000, Sigma Aldrich, D9542) for 5 min at RT. Cover slips were mounted with Fluoromount-G (Biozol, SBA-0100-01,) and sealed with clear nail polish after 24 h. Imaging was performed at Zeiss LSM780 confocal microscope using Zen black (2012 SP5) software (Carl Zeiss, Jena). Five regions per cover slip were selected and analyzed.

#### Morphological analysis

THP-1 cells were differentiated as described above and stimulated using LP-211 (Sigma Aldrich, SML1561-5MG, 10 µm) solved in DMSO or DMSO for control at day 3, respectively. Cell surface was stained using Cell Mask Plasma Membrane Stain (1:100, Life Technologies, C37608) in tyrode buffer according to manufacturer’s protocol. In short, on day 5 after seeding cells were washed with prewarmed PBS and incubated for 10 min at 37 °C with the staining solution. Cells were subsequently washed with PBS and imaged in tyrode buffer at Zeiss LSM780 confocal microscope (Carl Zeiss, Jena). Images of single cells with the main morphology (M0 – round, M1 – stretched, M2a – enlarged, M2c – enlarged) were separated using the MotiQ ImageJ/Fiji plugin [33, 34]. Quantification of shape and protrusions was performed in ImageJ/Fiji by manually drawing segmented lines and using the inbuild “measure” tool for measuring length and numbers. All values < 1 μm were excluded from analysis.

### Scratch assay

For the scratch assay, 2 × 10^5^ cells per 48 well were seeded and differentiated as described above. On day5, medium was renewed and cells were stimulated using LP-211 (10 µm, in DMSO) or DMSO (10%), respectively. The confluent cell layer was scratched in a vertical line using a p200 pipet tip. Recovery of wounded area was documented using Zeiss Axio Oberver.Z1/7, with Zen 3.2 Blue software (Carl Zeiss, Jena). For quantification of the recovered area, Fiji Wound healing size tool was modified [35]. Experiments with recovered areas less than the mean recovered area of M2c-like macrophages under control stimulation were excluded from analysis.

### Phagocytosis assay

A phagocytosis assay was performed as described in Sieve et al. [32]. In short, THP-1 and CD14^+^ cells were differentiated and stimulated with LP-211 (10 µm, in DMSO) or DMSO (100%) for control on day 3 or 5 after seeding. The inhibitors ZCL278 (Tebubio, T1855, 50 µm solved in DMSO), Y-27632 (Merck, 688000, 10 µm solved in DMSO), SQ22536 (MedChemExpress, HY-100396, 100 µm solved in DMSO) were added 30 min prior LP-211 (10 µm, in DMSO) treatment in THP-1-derived M1-like macrophages to analyze the impact of different signaling pathways. Cells were stimulated again after 24 h and subsequently incubated with 1 × 10^6^ particles of Zymosan A (*S. cerevisiae* BioParticles™-Texas Red™ conjugate, ThermoFischer, Z2843). On day 5 or 7 after seeding, cells were incubated with Hoechst 33342 (1:2000 in culture medium, Invitrogen, H3570) for 15 min at 37 °C and washed twice with prewarmed PBS. Fluorescence intensities were measured at Cytation5 image reader (BioTek) and quantified with Gene5 Image Prime 3.11 software (BioTek). Relative phagocytosis rate was calculated automatically by the software.

### Multiplex assays of THP-1-derived macrophages

On day 5 after seeding and differentiation, THP-1-derived macrophages were changed into plain medium and treated with DMSO or LP-211 (10 µm solved in DMSO) respectively. After 24 h, on day 6, the stimulation was renewed. On day 7, supernatant was collected, centrifuged at 1000 g for 15 min at 4 °C and stored at -80 °C until measurement. Supernatants were analyzed using Bio-Plex Pro Human Inflammation Panel 1, 27-Plex (BioRad, M500KCAF0Y) with the Bio-Plex 200 system (Bio-Rad). For normalization, cells were lysed as described above for Western Blot and Pierce BCA Protein Assay Kit (ThermoFisher scientific, 23225) was used to determine protein concentrations. Measured chemokine and cytokine concentrations were normalized to corresponding protein concentration of the sample. Analysis was performed on values within the standard range of each analysis. Analytes with one value were excluded from the analysis. Principle component analysis was performed using GraphPad Prism version 11. Analytes with values below the lowest value of the standard were include with the half minimal concentration of each standard [36].

### Statistical analysis

The presented data are shown as mean ± standard deviation (SD). Statistical analysis was performed using GraphPad Prism version 11. All data were subjected to outlier analysis and normal distribution tests. Applied statistical tests are indicated in the respective figure legends.

## Results

### Differentiation of THP-1 monocytes in different subtypes

**Fig. 1:**
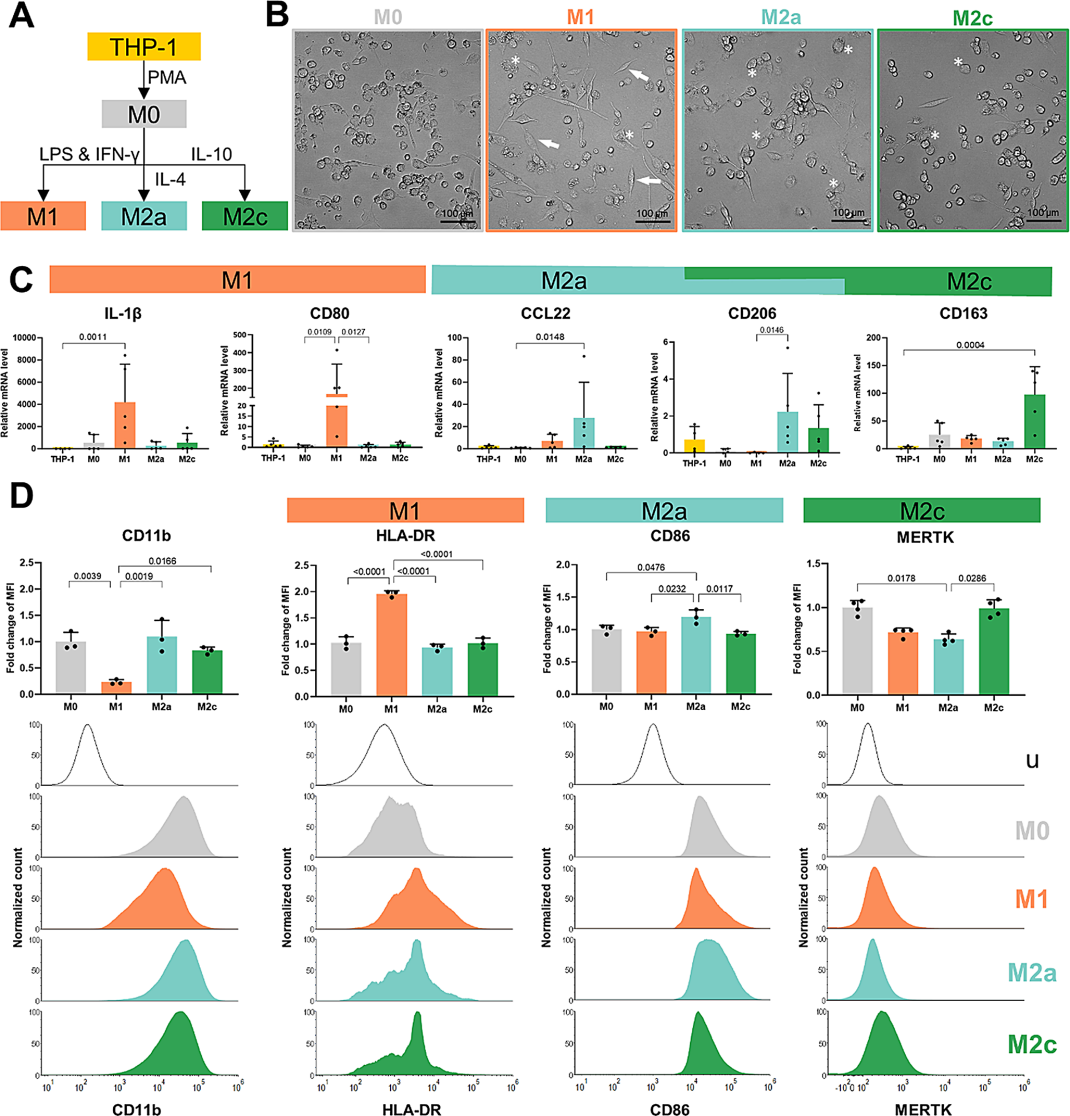
Basal characterization of THP-1-derived macrophage subtypes. **A**: Differentiation scheme of THP-1-monocytes into M0-, M1-, M2a-, and M2c-like macrophages. **B**: Representative morphology of differentiated macrophages, arrow indicates stretched cells, * highlights enlarged cells. **C**: mRNA expression levels of macrophage markers. Statistical significance was evaluated using Kruskal-Wallis with Dunn’s multiple comparison test (*N* = 5 independent differentiations). **D**: Quantitative analysis of surface marker expression of CD11b, HLA-DR, CD86, and MERTK for each macrophage subtype using flow cytometry. Fluorescence intensity shifts are displayed with normalized counts; u: unstained control. Fold changes of median fluorescence intensities (MFI) relative to M0-like macrophages were analyzed using ordinary one-way ANOVA with Tukey’s multiple comparisons test (CD11b, HLA-DR, CD86) and Kruskal-Wallis with Dunn’s multiple comparison test (MERTK) (*N* ≥ 3 independent differentiations)

First, we optimized the protocol for differentiation of THP-1 cells to defined subtypes of macrophages (Fig. 1A). To this end, THP-1 monocytes were treated with PMA, which results in cell adherence and generation of precursor M0-like macrophages. To polarize specific macrophage subtypes, cells were treated with LPS and interferon gamma (IFN-γ) to induce M1-like macrophages. Treatment of M0-like macrophages with interleukin 4 (IL-4) or IL-10 leads to either M2a- or M2c-like macrophages, respectively [30]. All generated macrophage subtypes displayed a distinct morphology [37]: the majority of M0 cells were small and round; M1-like stretched and enlarged; M2a- and M2c-like enlarged (Fig. 1B). To confirm successful differentiation, we verified expression of mRNA encoding particular macrophage markers (Fig. 1C). Pro-inflammatory M1-like macrophages showed significantly increased expression of IL-1β as well as CD80. Differentiation into M2a-like macrophages was confirmed by increased expression of CCL22 and CD206, whereas M2c-like macrophages demonstrated higher expression of CD163. In addition, M2c-like macrophages also expressed CD206, which is a broad M2 macrophage marker.

To further verify proper macrophage polarization, we analyzed expression of defined cell surface markers using flow cytometry analysis (Fig. 1D and Fig. S1). All subtypes of macrophages displayed a shift in median fluoresce intensity (MFI) for pan-macrophage marker CD11b (Fig. 1D). Furthermore, M1-like macrophages showed enhanced levels of the HLA-DR. In contrast, the percentage of CD86^+^ cells were significantly increased in M2a-like macrophages, while M2c-like macrophages demonstrated significantly elevated levels of proto-oncogene tyrosine-protein kinase MER (MERTK) on the cell surface compared to M2a-like macrophages. Taken together, this combined analysis confirms that our optimized differentiation protocol leads to generation of robust subsets of macrophages.

### Expression of 5-HT7R in different macrophage subtypes

**Fig. 2:**
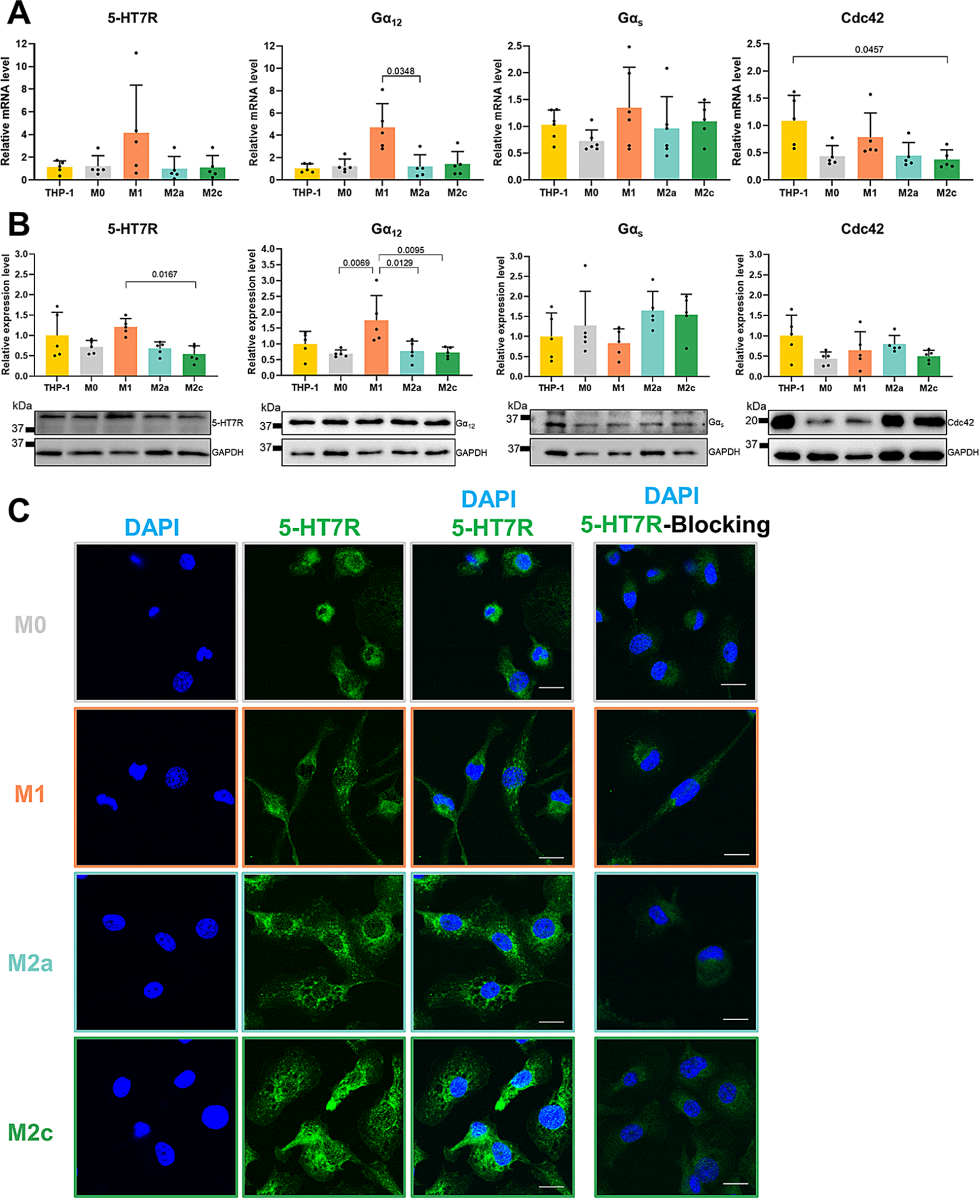
THP-1-derived macrophages express the 5-HT7R and downstream signaling molecules. **A**: mRNA levels of 5-HT7R and Gαs, Gα12, and Cdc42. Statistical difference was evaluated using Kruskal-Wallis with Dunn’s multiple comparison test (*N* ≥ 5). **B**: Protein expression levels of 5-HT7R and its downstream effectors shown by western blot. Statistical analysis was performed using ordinary one-way ANOVA with Tukey’s multiple comparisons (5-HT7R, Gα12) and Kruskal-Wallis with Dunn’s multiple comparison (Gαs, Cd42) test (*N* = 5). **C**: Representative images visualizing 5-HT7R localization. Antibody specificity was confirmed with a corresponding blocking peptide (right panel; scale bars 20 μm)

It has been suggested that variable expression of defined 5-HT receptors can be responsible for different effects of serotonin on immune cells [12, 14]. Therefore, we next analyzed the expression of the 5-HT7R as well as its down-stream effectors in different subsets of THP-1-derived macrophages. Results of quantitative real-time PCR (qRT-PCR) revealed a tendency for increased amount of mRNA encoding 5-HT7R and Gα12 protein in M1-like macrophages compared to monocytes and M2-like macrophages, while Gαs mRNA levels were similar in all groups (Fig. 2A). Of note, expression of mRNA encoding the small GTPase Cdc42 was reduced upon differentiation from THP-1 monocytes to macrophages showing significantly lower levels in M2c-like macrophages (Fig. 2A). Because the level of mRNA transcripts does not necessarily correlate with the protein expression level, we next analyzed protein expression of 5-HT7R and its signaling components by western blot. In line with transcript levels, expression of 5-HT7R was visible in all cell types (Fig. 2B). Of note, expression of 5-HT7R and the Gα12 subunit was significantly increased in M1-like macrophages compared to M2-like macrophages and to monocytes and M2-like macrophages, respectively. Immunocytochemical analysis further confirmed expression of 5-HT7R within the intracellular compartments as well as at the plasma membrane in all THP-1-derived macrophages (Fig. 2C). In these experiments, antibody specificity was confirmed using a specific blocking peptide (Fig. 2C, right panel). Thus, these combined data demonstrate that THP-1-derived macrophages represent a suitable model to study the functional implication of 5-HT7R-mediated signaling in regulation of macrophage functions.

### Stimulation of 5-HT7R modulates the morphological profile of THP-1-derived macrophages

**Fig. 3:**
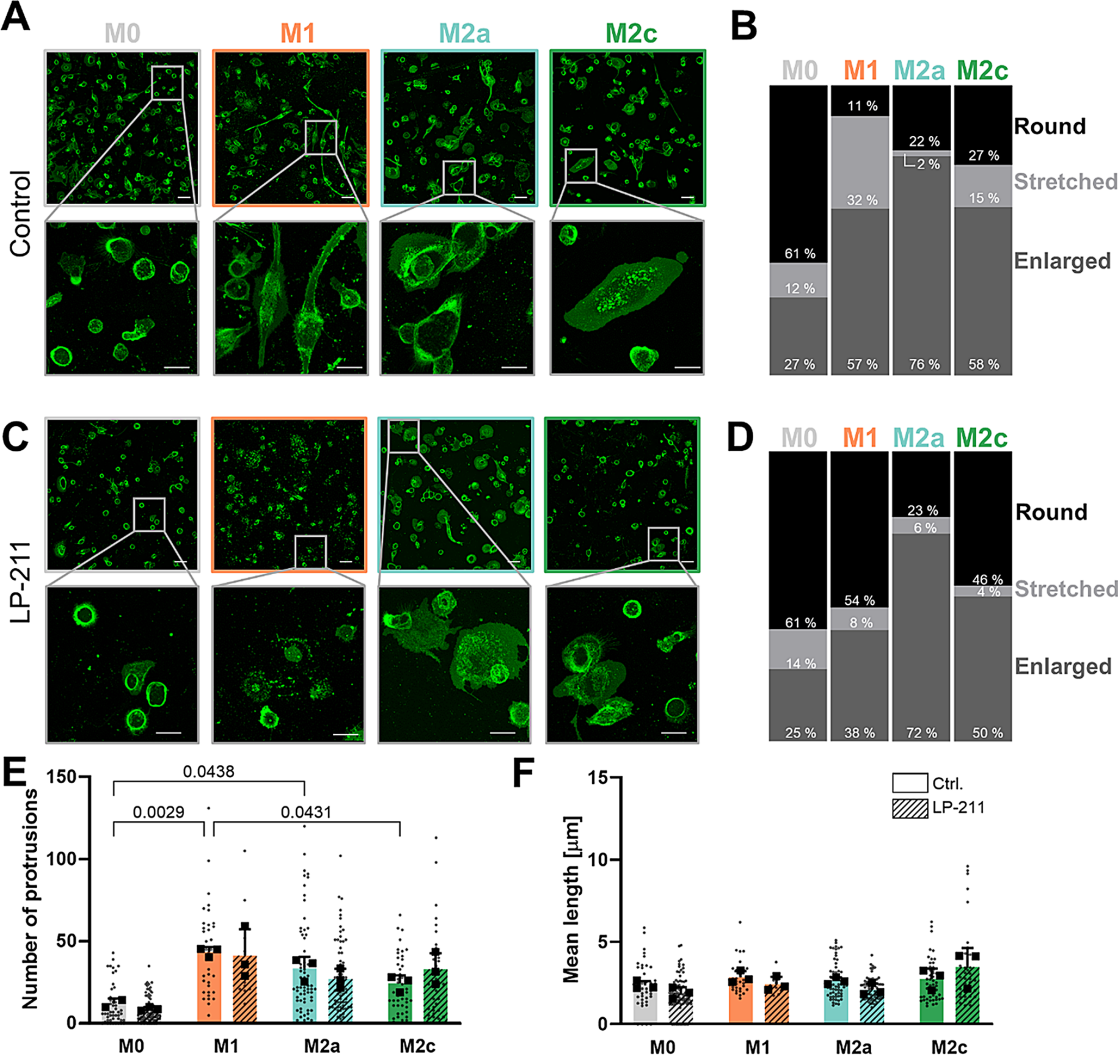
5-HT7R activation influences macrophage morphology. **A**: Characterization of macrophages morphology using plasma membrane staining in living cells. Representative images of cell shape on day 5 of differentiation are shown. Scale bars 50 μm in upper panel and 20 μm in magnification. **B**: Relative quantification of cell shape by counting round, stretched and enlarged phenotypes after THP-1 differentiation. **C**: Representative images of THP-1-derived macrophage morphology following LP-211 treatment (10 µm; 48 h). Scale bars 50 μm in upper panel and 20 μm in magnification (*N* = 3). **D**: Quantification of morphology profiles after treatment with LP-211 (10 µm; 48 h). **E**: Quantification of protrusion numbers in differentiated macrophages without and after LP-211 (10 µm; 48 h) treatment. Statistical analysis was performed on daily mean values (*N* = 3) using two-way ANOVA with Tukey’s multiple comparisons test. **F**: Analysis of mean length of protrusions under basal conditions and after 5-HT7R stimulation with LP-211 (10 µm; 48 h). Statistical analysis was performed on daily mean values (*N* = 3) using two-way ANOVA with Tukey’s multiple comparisons test

Cell morphology represents an essential feature for various macrophage functions including migration, phagocytosis, and cell-cell interactions. Therefore, we characterized basal cell morphology after macrophage differentiation using plasma membrane staining followed by live cell imaging (Fig. 3A-B). The majority of M0-like macrophages were rounded (61%) with only 27% displaying an enlarged morphology. In contrast, the latter was a dominant cell form for both M1- and M2-like macrophages (57% for M1-like, 76% for M2a-like and 58% for M2c-like; Fig. 3B). One particular feature of M1-like macrophages, in comparison to the other investigated monocytic cell types, was a substantially increased amount of elongated and enlarged cells (32%; Fig. 3B).

Pharmacological activation of the 5-HT7R by the selective agonist LP-211 (48 h) [38–41] results in a considerable decrease in number of elongated and enlarged cells in M1-like macrophages to 8% and 38%, respectively, while the fraction of rounded cells increased from 11 to 54% (Fig. 3C-D). Similar tendencies were also obtained in M2c-like macrophages, in which the percentage of rounded cells in relation to enlarged and elongated cells increased from 27 to 46% (Fig. 3C-D). In contrast, M0- and M2a-like macrophages show only minor morphological changes in response to 5-HT7R activation. Of note, LP-211 treatment did not influence the number or mean length of protrusions of any macrophage subtypes (Fig. 3E and F). However, detailed morphological analysis (Fig. S2) revealed that M0-like macrophages possess a reduced number of plasma membrane protrusions compared to other macrophage subsets, while the mean length of protrusions does not differ between all macrophage subtypes and after pharmacological treatment (Fig. 3E).

It is important to mention that morphological changes obtained after 5-HT7R stimulation were not mediated by the agonist-mediated shift in the differentiation profiles, since mRNA expression levels of differentiation markers in all macrophage subtypes did not change after LP-211 treatment (Fig. S3).

### 5-HT7R signaling selectively restrains migratory capability of pro-inflammatory macrophages

**Fig. 4:**
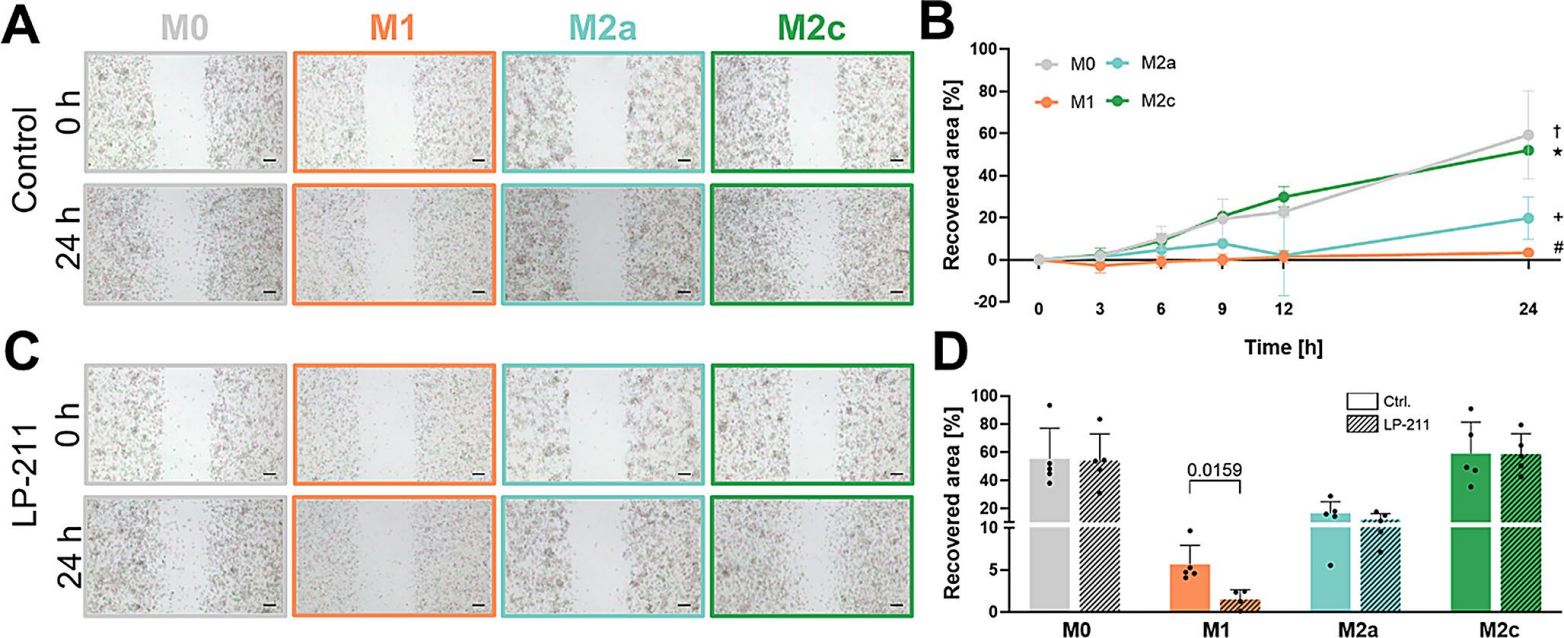
Motility of macrophages is influenced by 5-HT7R activity. **A**: Representative images of scratch area at beginning (0 h) and 24 h after scratch initiation for all macrophage subtypes. Scale bars 200 μm. **B**: Recovered area of macrophage subtypes relative to scratch area at 0 h. Quantification of migratory ability after 24 h. Data are presented as mean with standard deviation. Adjusted p values: † M0 vs. M1 *p =* 0.0004, ^+^ M0 vs. M2a *p =* 0.0084, ^#^ M1 vs. M2c *p* = 0.002, and M2a vs. M2c *p =* 0.0398 using one-way ANOVA with Tukey’s multiple comparisons test (*N* = 4). **C**: Images of scratch area at 0 h and 24 h after LP-211 treatment (10 µm). Scale bars 200 μm. **D**: Quantification of macrophage migration ability after 24 h after LP-211 (10 µm) or control treatment. Statistical significance was evaluated using unpaired Mann-Whitney test between control and LP-211 conditions of each macrophage subtype (*N* = 5)

To assess the migratory ability of differentiated macrophage subtypes, we applied a wound healing scratch assay. Under basal conditions, M0- and M2c-like macrophages exhibited the highest recovery rate of the scratched area reaching 59.3% and 52.1% after 24 h, respectively, followed by M2a-like macrophages (19.8% after 24 h). In contrast, pro-inflammatory M1-like macrophages demonstrated the lowest migratory ability at all analyzed time points with only 3.3% recovery at the end point (Fig. 4A-B). Stimulation of 5-HT7R with LP-211 significantly slowed the migratory ability of M1-like macrophages to about 1.5% recovered area after 24 h (Fig. 4C-D). In contrast, the migratory properties of M0-like and M2-like macrophages remained unaffected upon pharmacological 5-HT7R activation (recovered area of 54.0%, 12.1%, and 58.9% for M0-like, M2a-like and M2c-like macrophages after 24 h, respectively; Fig. 4C-D). Taken together, these results suggest that 5HT7R-mediated signaling plays an important role in regulation of morphology and motility of pro-inflammatory macrophages.

### Phagocytosis rate of pro-inflammatory macrophages is reduced upon 5-HT7R activation

**Fig. 5:**
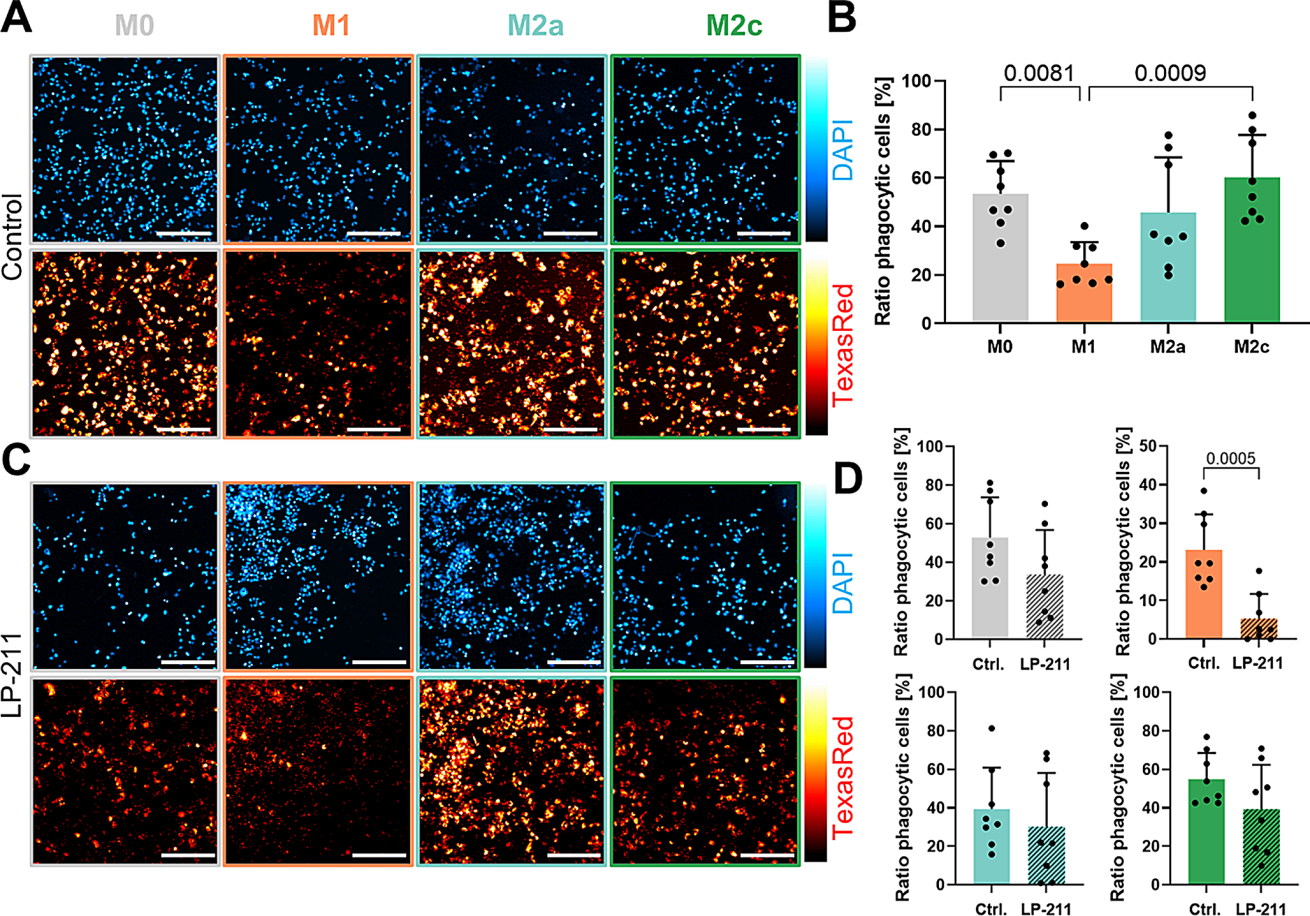
Phagocytosis rate of M1-like macrophages is reduced upon 5-HT7R activation. **A**: Representative images of DAPI stained nuclei (upper row) and Texas red-coupled Zymosan A particles (lower row) in control-treated macrophages. Scale bars 200 μm. **B**: Fraction of phagocytic macrophages showing engulfment of Texas red-coupled Zymosan A particles. Statistical differences are evaluated using ordinary one-way ANOVA with Tukey’s multiple comparison test (*N* = 8; each point displays the mean of 3 replicate wells). **C**: Representative images of macrophages treated with LP-211 (10 µm) showing DAPI and Texas red-coupled Zymosan A particle fluorescence after phagocytosis assay. Scale bars 200 μm. **D**: Quantification of phagocytosis rate of macrophage subtypes with control or LP-211 treatment (10 µm; 48 h). Statistical significance was determined using unpaired two-tailed t-test (*N* = 8; each point displays the mean of 3 replicate wells)

Phagocytosis represents the essential function of macrophages, enabling the clearance of pathogens, cell debris and maintenance of tissue integrity. To quantitatively validate the phagocytosis rate of THP1-deriverd macrophages, we exposed the cells to fluorescently labelled Zymosan A particles (derived from *Saccharomyces cerevisiae*) followed by real-time visualization of phagocytic events using fluorescence microscopy. As shown in Fig. 5A-B and Fig. S4 A, M0-, M2a- and M2c-like macrophages exhibit relatively high phagocytosis rates (M0- 53.4%, M2a- 45.7%, M2c-like 60.3% of cells show engulfed particles after 24 h), and pharmacological activation of the 5-HT7R by LP-211 for 48 h has no effect on their phagocytic properties (Fig. 5C-D). In contrast, the number of M1-like pro-inflammatory macrophages, which were able to phagocytose Zymosan A, was significantly lower already under basal conditions (24.6%; Fig. 5A-B). Noteworthy, treatment of M1-like macrophages with LP-211 drastically reduced their phagocytosis rate by 4.5-fold, reaching 5.3% (Fig. 5C-D). This demonstrates that 5-HT7R-mediated signaling exclusively reduces phagocytic activity of THP-1-derived pro-inflammatory M1-like macrophages.

To determine, which signaling pathway might be responsible for the diminished phagocytosis of pro-inflammatory M1-like macrophages after LP-211 stimulation, we repeated the phagocytosis assay in THP-1-derived M1-like macrophages in the presence of various compounds that modulate components of the 5-HT7R signaling pathways. Specifically, we tested (i) the Cdc42 inhibitor ZCL278, (ii) the Rho-Kinase (ROCK) inhibitor Y-27632, and (iii) the adenylyl cyclase (AC) inhibitor SQ22536. Treatment with SQ22536 (100 µm, 48 h), Y-27632 (10 µm, 48 h), and ZCL278 (50 µm, 48 h) did not alter the basal phagocytic ability of THP-1-derived macrophages (Fig. S4B). More importantly, when combined with LP-211, none of these inhibitors prevented the LP-211-induced reduction in the ratio of phagocytic M1-like macrophages. These data suggest that the reduction in phagocytosis induced by LP-211 in M1-like macrophages does not rely on the 5-HT7R canonical signaling pathways, implying the involvement of alternative signaling mechanisms.

### Stimulation of 5-HT7R modulates cytokine and chemokine secretion

**Fig. 6:**
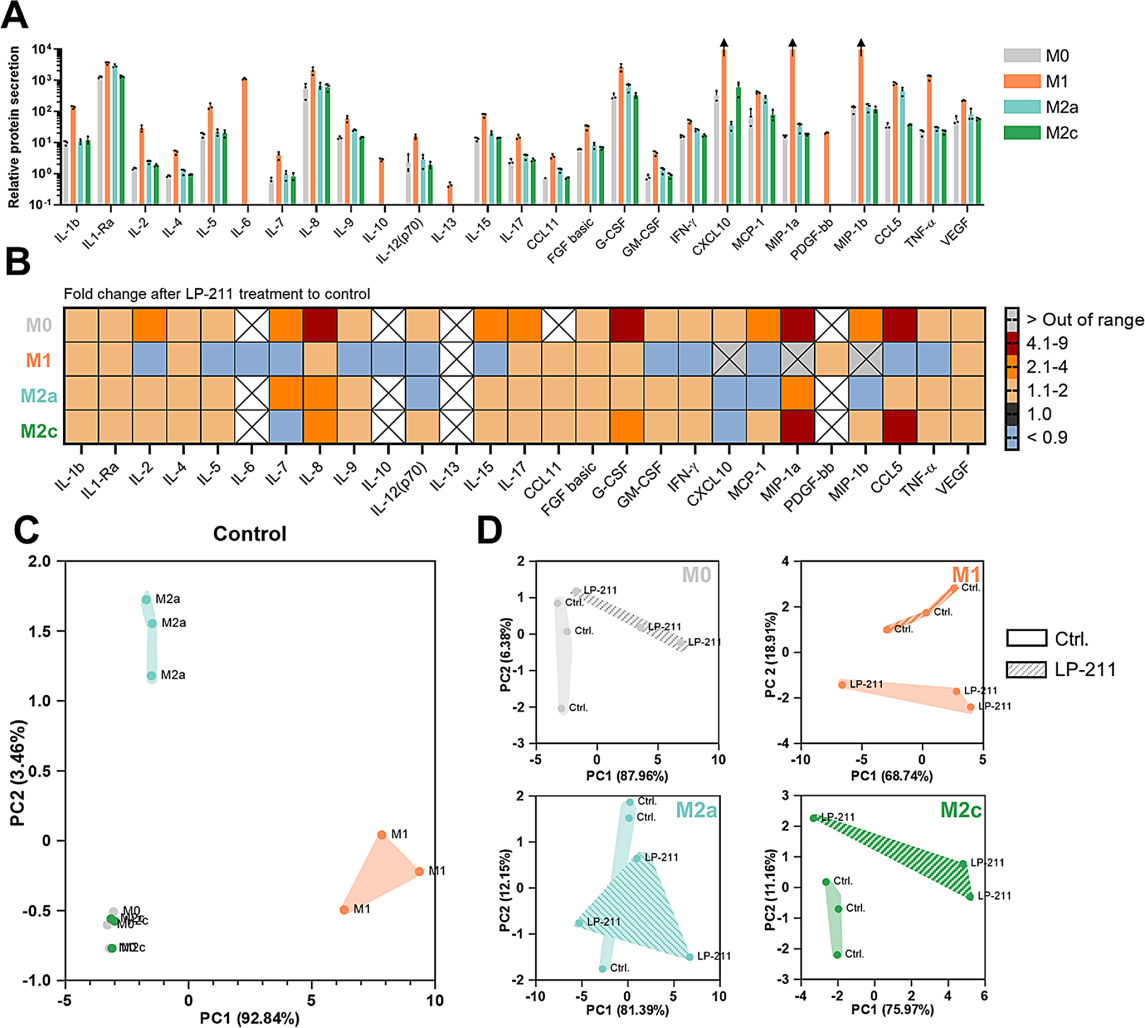
Modulation of secretion ability of macrophages upon 5-HT7R stimulation. **A**: Relative secretion profile in the supernatant of THP-1-derived macrophages by normalization to protein concentration (*N*=3). Arrows indicate concentrations above the highest value of the standard. **B**: Relative fold changes of secreted proteins after LP-211 (10 µm) treatment starting day 3 after seeding compared to control. **C**: Principal component analysis of three independent differentiations (one dot displays one differentiation) using the first two principal components (*N*=3). **D**: Principal component analysis to visualize clustering under basal conditions and after LP-211 treatment for each macrophage subtype. Each principal component was assessed in a separated analysis using Kaiser’s rule (*N*=3)

The process of wound healing is highly dependent on the composition of cytokines and chemokines secreted by present immune cells. To assess the impact of 5-HT7R-mediated signaling on cytokine and chemokine levels, we analyzed the secreted amounts of multiple cytokines and chemokines in the supernatants of THP-1-derived macrophages under basal conditions and after pharmacological 5-HT7R activation (Fig. 6). Under basal conditions, pro-inflammatory M1-like macrophages showed increased amounts of secreted proteins compared to M0 and M2-like macrophages (Fig. 6A). In particular, secretion of IL-1Ra, IL-8, CCL5, granulocyte-colony-stimulating factor (G-CSF) and tumor necrosis factor-α (TNF-α) was substantially higher in comparison to M0-, M2a-, and M2c-like macrophages. These pro-inflammatory cytokines and chemokines are of primary importance for leukocyte recruitment, their differentiation and the initiation of an inflammatory response [42–44]. Interestingly, only M1-like macrophages secreted the interleukins-6 and -13 within the detection range. The IL-13 possess predominantly anti-inflammatory properties, whereas IL-6 acts pro-inflammatory but has also been shown to facilitate the differentiation of M2-like macrophages [45, 46].

Treatment of the different subtypes of macrophages with the 5-HT7R agonist LP-211 results in heterogeneous effects (Fig. 6B): Secretion of IL-1Ra, IL-8, and G-CSF by pro-inflammatory M1-like macrophages was similar to basal conditions. Noteworthy, LP-211 treatment results in remarkable (up to 8-fold) increase in secretion of IL-8, G-CSF, macrophage inflammatory protein 1-α (MIP-1a), and CCL5 by M0- and M2c-like macrophages in comparison to each control condition. Interestingly, pro-inflammatory M1-like macrophages respond to LP-211 treatment with reduced secretion of most of the analyzed proteins.

To gain deeper insight into the molecular mechanisms underlying 5-HT7R-mediated changes in cytokine secretion, we analyzed the mRNA expression levels of cytokines whose secretion was modulated by LP-211 treatment. Under basal conditions, the mRNA expression profile of THP-1-derived macrophages correlated with the cytokine levels measured in the cell supernatants (Fig. S5). However, despite modulation of cytokine secretion by LP-211, treatment had no effect on the mRNA expression levels of these cytokines (Fig. S5). These results suggest that the effects of LP-211 are not mediated by changes in cytokine transcription, but rather by alternative mechanisms, such as cytokine processing, maturation, or release.

We also performed a principal component analysis of the secreted proteins in the supernatant of three independent THP-1 macrophage differentiation experiment. To visualize clustering under basal conditions, two components representing 97% of the observed variation in the data set, were displayed. Principal component 1 (PC1) represents 93% whereas PC2 stand for 4% of the variation leading to a separate clustering of pro-inflammatory M1-like and anti-inflammatory M2a-like macrophages. M0 and M2c- like macrophages cluster together under basal conditions (Fig. 6C). Upon treatment with LP-211, M0-, M1-, and M2c-like macrophages respond with an individual shift in their secretion profiles (Fig. 6D).

### Influence of 5-HT7R-mediated signaling on macrophages derived from primary human CD14^+^ cells

**Fig. 7:**
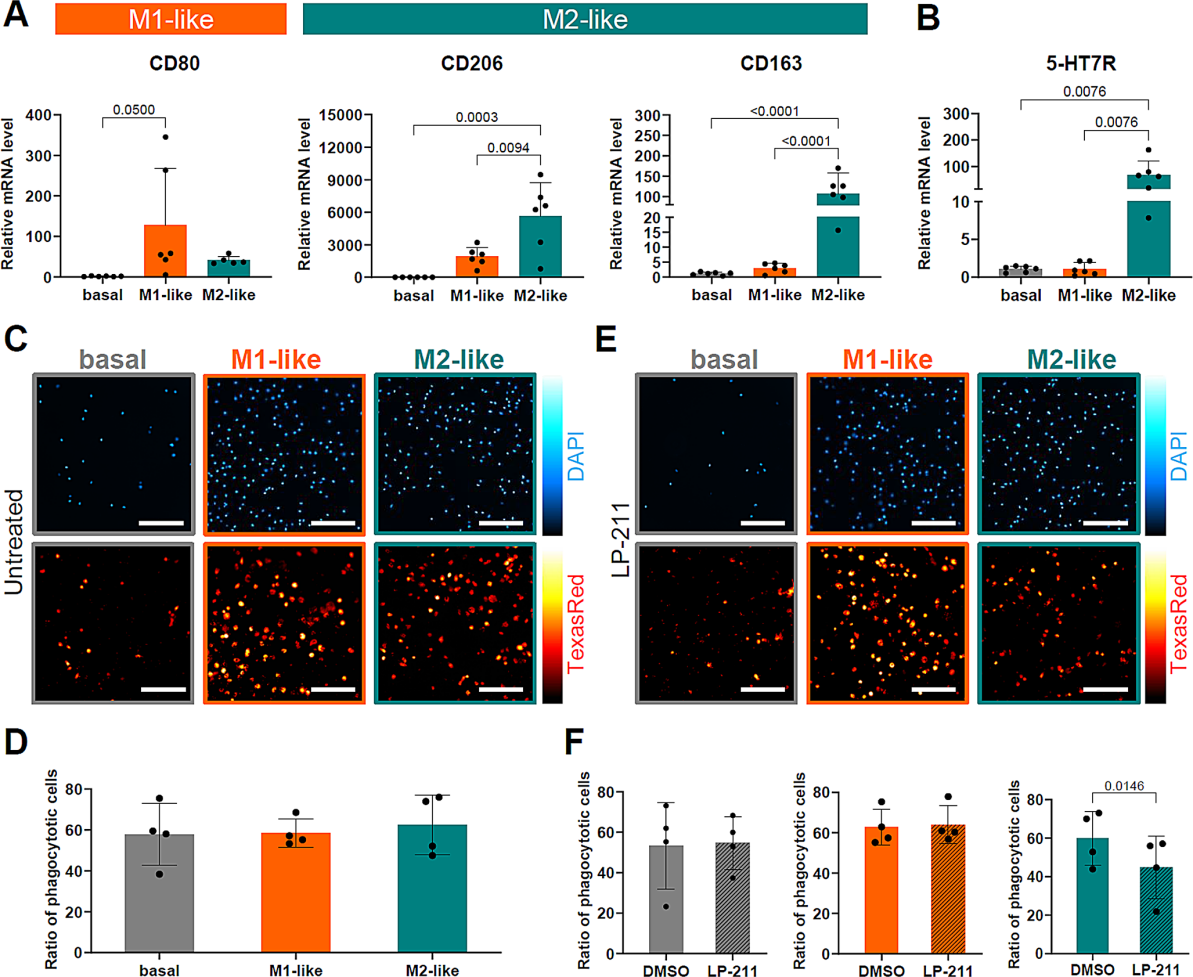
Impact of 5-HT7R signaling on primary human CD14^+^ macrophages. **A**: mRNA expression levels of differentiation markers in CD14^+^ cells. Statistical significance was evaluated using ordinary one-way ANOVA with Tukey’s multiple comparisons test (*N* = 6 independent differentiations/donors). **B**: mRNA expression level of 5-HT7R at day 7 of differentiation. Statistical difference was evaluated using ordinary one-way ANOVA with Tukey’s multiple comparisons test (*N* = 6). **C**: Representative images of DAPI stained nuclei (upper row) and Texas red-coupled Zymosan A particles (lower row) in CD14^+^-derived macrophages after 24 h phagocytosis assay under basal conditions. Scale bars 200 μm. **D**: Quantification of phagocytic cells showing engulfment of Texas red-coupled Zymosan A particles under basal conditions. Statistical differences were evaluated using ordinary one-way ANOVA with Tukey’s multiple comparison test (*N* = 4; each point displays the mean of ≥ 4 replicate wells). **E**: Representative images of macrophages treated with LP-211 (10 µM) showing DAPI and Texas red-coupled Zymosan A particle fluorescence after phagocytosis assay. Scale bars 200 μm. **F**: Quantification of phagocytosis rate of macrophage subtypes with control (DMSO) or LP-211 treatment (10 µM; 48 h). Statistical significance was determined using paired two-tailed t-test (*N* = 4; each point displays the mean of ≥ 4 replicate wells)

While THP-1 cells are widely used as a reliable in vitro model to study the basic mechanisms of monocyte/macrophage biology [25, 32, 47, 48], they have notable limitations. To address this, we established primary human CD14^+^ blood cells as an additional model system. These cells were differentiated into M1- and M2-like macrophage subtypes using treatment with GM-CSF and M-CSF, respectively. Successful differentiation was confirmed by qRT-PCR using CD80 as a marker for M1-like macrophages, while CD206, and CD163 were used as markers for anti-inflammatory M2-like macrophages (Fig. 7A).

We also assessed the expression of 5-HT7R at various stages of differentiation (Fig. S6 A) and observed a significant increase in 5-HT7R mRNA expression levels specifically in M2-like macrophages (Fig. 7B). Furthermore, we found that M2-like macrophages exhibit high mRNA levels of key downstream effectors of the 5-HT7R signaling pathway, including Gα12, Gαs, and Cdc42 (Fig. S6 B). These findings demonstrate that M2-like macrophages are an appropriate model for investigating 5-HT7R-mediated signaling.

Next, we examined the phagocytic activity of basal CD14^+^ cells as well as M1- and M2-like macrophages. Under basal conditions, no significant differences in phagocytosis were observed among the different cell types (Fig. 7C-D). However, treatment with the 5-HT7R agonist LP-211 significantly reduced phagocytosis only in M2-like macrophages (Fig. 7E-F), which correlates with their high expression levels of 5-HT7R and its associated effectors. In contrast, LP-211 had no effect on basal CD14^+^ cells or M1-like macrophages reflecting very low levels of 5-HT7R expression in these cells. These results further support our previous findings and emphasize the important role of 5-HT7R in regulating human macrophage functions.

## Discussion

The monocytic cell line THP-1 can be differentiated into macrophage subtypes with specific functions [25]. Depending on applied differentiation agents and culture protocols, the obtained macrophage properties can substantially differ [25, 28]. Here we confirmed previous data that the polarization of THP-1-derived macrophages is reflected by specific mRNA and flow cytometry expression patterns as well as by typical cell morphology. Among others, IL-1β, CD80, and HLA-DR – established markers for pro-inflammatory macrophages - were upregulated in M1-like macrophages [28, 32, 49–51]. To distinguish between M2a- and M2c-like macrophages, we compared the expression of CD163, CD86, MERTK, and CD14, which displayed a clear differentiation between the macrophage subtypes. This data suggests that our differentiation protocol is suitable for detailed analysis of macrophage functions.

The morphology of macrophages changes upon differentiation status, and is important for exhibition of their function [52]. Here, M1-like macrophages were mainly stretched and enlarged, whereas M2-like cells exhibited enlarged phenotypes compared to M0-like macrophages. These phenotypes were also observed in human monocyte-derived macrophages (hMDMs) [37, 52]. Interestingly, mouse bone marrow derived macrophages (BMDM) possess different morphological phenotypes supporting existence of species-specific differences [53]. Stimulation of the 5-HT7R by LP-211 changed the morphology profile of cultured macrophages by increasing the population of round cells in M1- as well as M2a-like macrophages. Since specific morphological shapes of macrophages were shown to correlate with their activation status [54], LP-211 treatment might therefore modulate the activation status of these cells.

Migration is a crucial feature of macrophages allowing them to move towards the site of injury. This complex process involves cell attachment and detachment as well as actin cytoskeleton rearrangement with podosome formation [55]. Pro-inflammatory macrophages show reduced migration ability compared to anti-inflammatory macrophages. This drawback was described in different macrophage models with diverse chemoattractants [52, 56]. For example, it was shown that mouse M1-like macrophages express high levels of integrin α_D_β_2_ (CD11d/CD18), increasing their attachment, which in turn inhibits migration [56–59]. In addition, formation of podosomes as well as differences in actin cytoskeletal reorganization were observed between pro- and anti-inflammatory macrophages [37, 52]. In line with these observations, THP-1-derived M1-like macrophages in our study showed the weakest migration ability. More importantly, such weak ability for migration was further reduced upon 5-HT7R activation. This effect was accompanied by profound morphological changes – number of round cells was increased upon LP-211 treatment. This was in contrast to previous observation in dendritic cells, where 5-HT7R activation induced elongation of protrusions and enhanced migration, effects evoked by the G12 protein-mediated activation of small GTPases Rac1 and Cdc42 [15]. On the other hand, stimulation of 5-HT7R with LP-211 initiates Gs signaling leading to increased production of cAMP. This, in turn, could negatively modulate cell adhesion and migration via Epac-Rap1-mediated activation of integrins [60–62].

Phagocytosis is a central function of macrophages. The in vitro phagocytic efficacy of macrophages is influenced by the macrophages’ origin (tissue, species, cell line), differentiation agents (GM-/M-CSF, PMA, LPS, IFN-γ, IL-4, IL-13, IL-10) and used bioparticles (yeast, bacteria, coated beads) [63–65]. For example, application of differentiation reagents such as LPS and IFN-γ has been shown to differentially affect phagocytosis: treatment with LPS enhanced, while treatment with IFN-γ reduced phagocytic capacity of macrophages [66, 67]. It was also shown that M1-like macrophages have increased ability to phagocytose *S. aureus* bioparticles compared to other THP-1-derived macrophage subtypes [63]. Also Tedesco and co-workers demonstrated that M0- and M1-like macrophages displayed high levels of dextran-FITC phagocytosis, while M2-like macrophages showed reduced engulfment [50]. On the other hand, recent study by Hickman and co-workers demonstrated that M1-like hMDMs have significantly lower phagocytic capacity for Zymosan A bioparticles than M0- and M2- hMDMs [68]. Also in the present study, THP-1-derived pro-inflammatory M1-like macrophages showed very low phagocytic capacity of Zymosan A particles under basal conditions. More importantly, stimulation of 5-HT7R resulted in significantly reduced phagocytic ability particularly in this macrophage subtype. Of note, a similar inhibitory effect on phagocytosis was observed in primary human CD14^+^ cells differentiated into M2-like macrophages. These cells exhibited a significant increase in 5-HT7R mRNA levels, consistent with previous studies [19], as well as higher expression of its associated effectors, making M2-like macrophages an appropriate model for investigating 5-HT7R-mediated signaling.

What mechanism could underlie 5-HT7R-mediated regulation of phagocytosis? One essential step in phagocytosis is the remodeling of the actin cytoskeleton, which is accompanied by local changes in cAMP concentrations near the cell membrane [64, 69–72]. Both elevated cAMP and Epac1 levels have been shown to reduce phagocytic capacity [73–78]. However, our data indicate that the LP-211-induced reduction in phagocytosis is not dependent on receptor-mediated activation of AC, suggesting the involvement of alternative signaling pathways. One possible mechanism is the 5-HT7R-mediated activation of the mammalian target of rapamycin (mTOR) via Gα12 signaling, which has been shown to reduce phagocytic activity [79–81]. This pathway may contribute to the observed reduction in phagocytosis following LP-211 treatment. Future studies will be needed to investigate and verify this hypothesis.

Several studies have shown that 5-HT can modulate monocytes’ and macrophages’ secretory profile. Pretreatment with 5-HT leads to reduced mRNA levels of the cytokines and chemokines CXCL10, CXCL11, IDO, RSAD2, IL-27, IFIT2, and CXCL11 in hMDMs [20]. Beside this, 5-HT has been shown to induce a M2-like phenotype in macrophages by modulating gene expression profiles and cytokine production through 5-HT7R signaling [19]. Furthermore, 5-HT7R activation upregulates the release of IL-1β, IL-6, IL-12p40 and IL-8/CXCL8 and downregulates LPS-induced TNF-α release in human primary monocytes [17]. In this study, we found that under basal conditions pro-inflammatory M1-like macrophages secreted high levels of cytokines and chemokine compared to M0- and M2-like macrophages, which is in accordance to the results obtained in hMDMs [68, 82]. Pharmacological activation of the 5-HT7R with LP-211 boosts secretion of chemokines and cytokines particularly from M0- and M2-like macrophage subtypes. In particular M0-like macrophages showed increased secretion of pro-inflammatory cytokines IL-8, MIP-1α, G-CSF, and CCL5 (RANTES). These cytokines are important for neutrophil recruitment and migration of leukocytes, indicating a possible impact on early inflammatory responses [83–86]. In contrast, in M1-like macrophages LP-211 treatment resulted in slightly reduced secretion when compared to control conditions.

It is important to emphasize that the effects of LP-211 were not driven by changes at the transcriptional level but rather by alternative mechanisms. These mechanisms likely involve cytokine processing, maturation, or release. In macrophages, cytokine secretion is thought to follow the constitutive secretory pathway, wherein cytokines are not stored temporarily but are instead continuously released. The rate of this release is regulated by adjustments in post-Golgi trafficking [87, 88]. Given that LP-211 did not influence cytokine transcription in THP-1 cells, we hypothesize that its effects may involve disruption of post-Golgi trafficking processes, specifically those governing the transport of cytokines to the plasma membrane.

Taken together, we demonstrated that stimulation of the 5-HT7R selectively reduces THP-1-derived pro-inflammatory M1-like macrophages’ migratory and phagocytic properties. Furthermore, 5-HT7R activation shifts morphology profiles and alters M0-, M2a-, and M2c-like macrophages’ secretion properties. Thus, 5-HT7R-mediated signaling might be a new access point for the modulation of macrophage responses in the treatment of inflammatory diseases.

## Electronic supplementary material

Below is the link to the electronic supplementary material.

Supplementary Material 1

## Data Availability

The data that support the findings of this study are available from the corresponding author upon reasonable request.

## Abbreviations

5-HT: Serotonin
5-HT7R: Serotonin receptor 7
AC: Adenylyl cyclase
BMDM: Bone marrow derived macrophages
cAMP: Cyclic adenosine monophosphate
Cdc42: Cell division control protein 42 homolog
DMSO: Dimethyl sulfoxide
G-CSF: Granulocyte-colony-stimulating factor
GM-CSF: Granulocyte macrophage colony-stimulating factor
HLA-DR: Leukocyte antigen-DR isotype
hMDMs: Human monocyte-derived macrophages
IFN-γ: Interferon gamma
IL: Interleukin
LPS: Lipopolysaccharide
M-CSF: Macrophage colony-stimulating factor
MERTK: Proto-oncogene tyrosine-protein kinase MER
MFI: Median fluoresce intensity
MIP-1α: Macrophage inflammatory protein 1-α
mTOR: Mammalian target of rapamycin
PBMC: Peripheral blood mononuclear cells
PC: Principal component
PFA: Paraformaldehyde
PKA: Protein kinase A
PMA: Phorbol-12-myristat-13-acetat
qRT-PCR: Quantitative real-time PCR
ROCK: Rho-kinase
RT: Room temperature
SD: Standard derivation
TNF-α: Tumor necrosis factor-α
