# Supplementary material for: Serotonin receptor 5-HT7 modulates inflammatory-associated functions of macrophages
Source: Cell Mol Life Sci. 2025 Jan 21;82(1):51. doi: 10.1007/s00018-024-05570-z (PMC11747067; doi:10.1007/s00018-024-05570-z)
Supplement: Supplementary file 1 — Supplementary Material 1 [file 18_2024_5570_MOESM1_ESM.docx]

**Supporting Information**

**Serotonin receptor 5-HT7 modulates inflammatory-associated functions of macrophages**

Frauke S. Bahr^#^, Franziska E. Müller^#^, Martina Kasten, Nils Benen, Irina Sieve, Michaela Scherr, Christine S. Falk, Denise Hilfiker-Kleiner, Melanie Ricke-Hoch^ƚ^, Evgeni Ponimaskin^ƚ*^

Corresponding author:

Evgeni Ponimaskin

Hannover Medical School, Cellular Neurophysiology, Hannover, Germany
Ponimaskin.evgeni@mh-hannover.de, ORCID 0000-0002-4570-5130


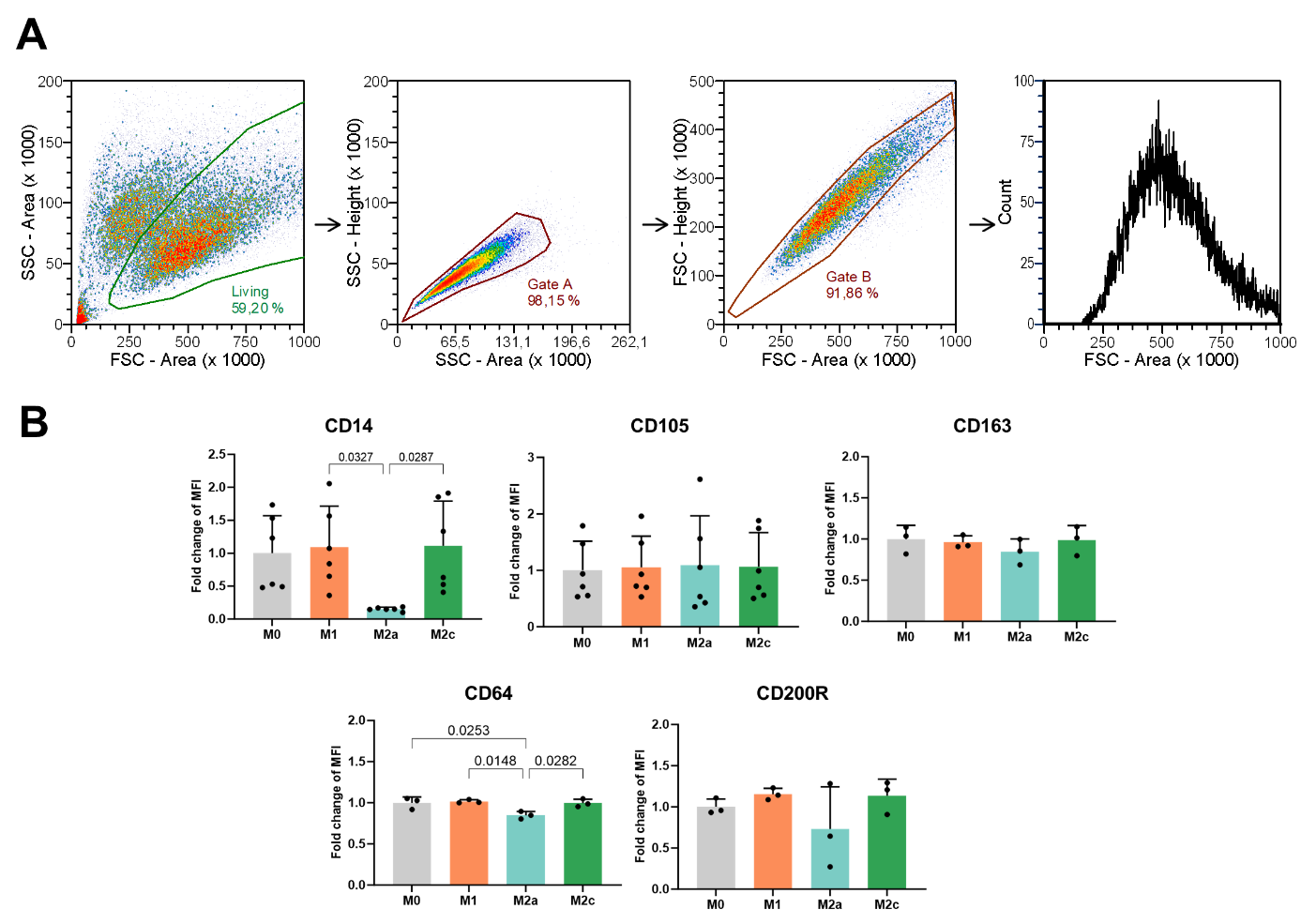


**Fig. S1** **Gating strategy for flow cytometry analysis**

**A:** Gating strategy during flow cytometry experiments. In a first step all living cells were selected. Second, doublet discrimination was performed (gate A and B). For further analysis, the relative count over FSC area was plotted. **B:** Fold changes of median fluorescence intensities (MFI) relative to M0-like macrophages. Analysis using ordinary one-way ANOVA with Tukey’s multiple comparisons test (N≥3 independent differentiations).


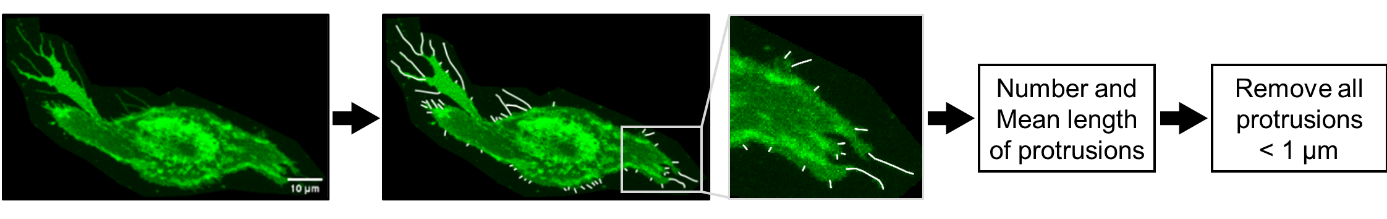


**Fig. S2** **Schematic scheme of protrusion analysis**

Step-by-step strategy for protrusion analysis using ImageJ software. Representative macrophage is shown. Scale bar, 10 µm.


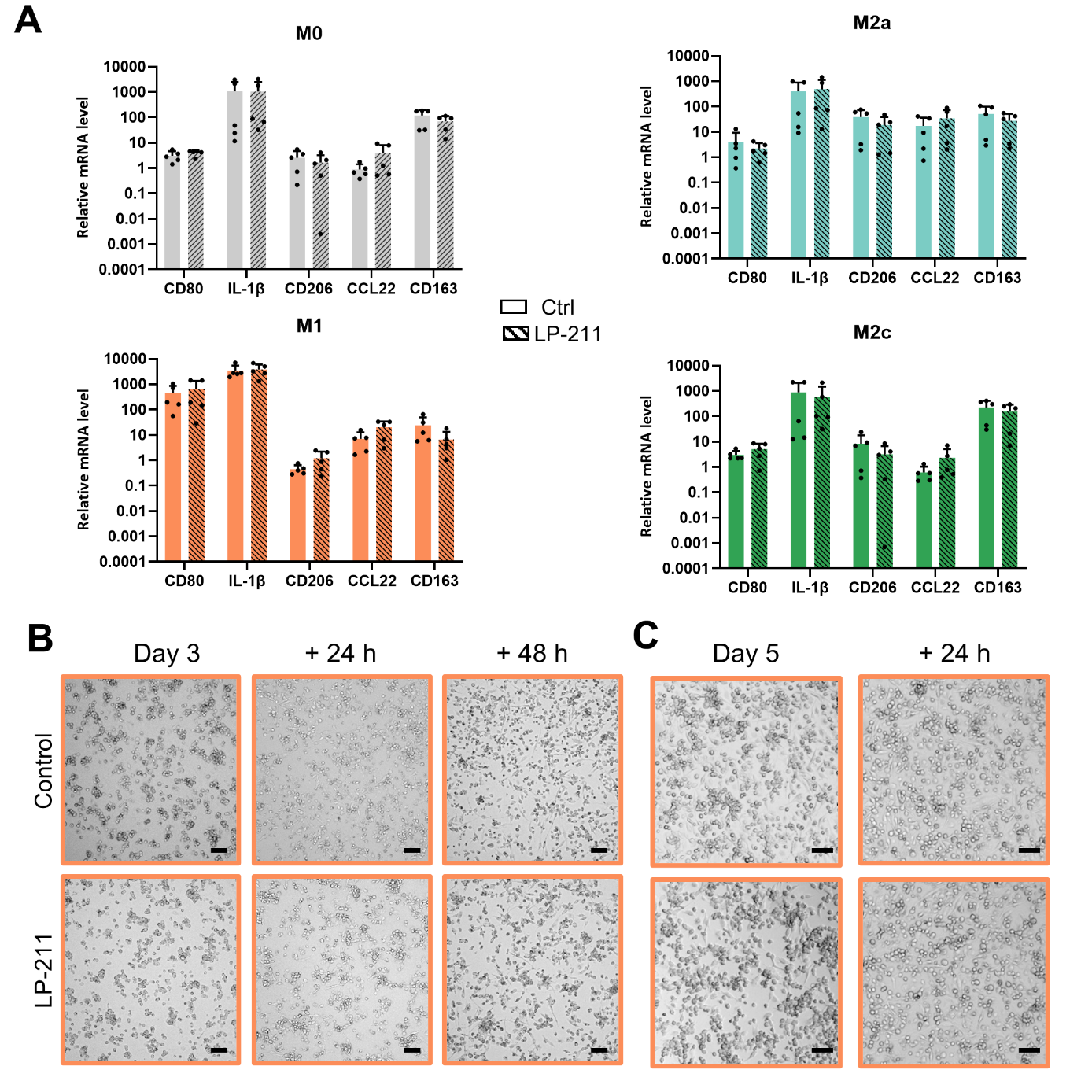


**Fig. S3 Pharmacological activation of 5-HT7R does not change macrophage differentiation marker expression or cell attachment**

**A:** Relative changes of macrophage differentiation markers between control and LP-211 treated cells (N≥5). **B:** Attachment of M1-like macrophages under control and LP-211-treated conditions after 0, 24 and 48 h. Scale bars 100 µm. **C:** M1-like macrophage following LP-211 (10 µm) and control. Scale bar 100 µm.


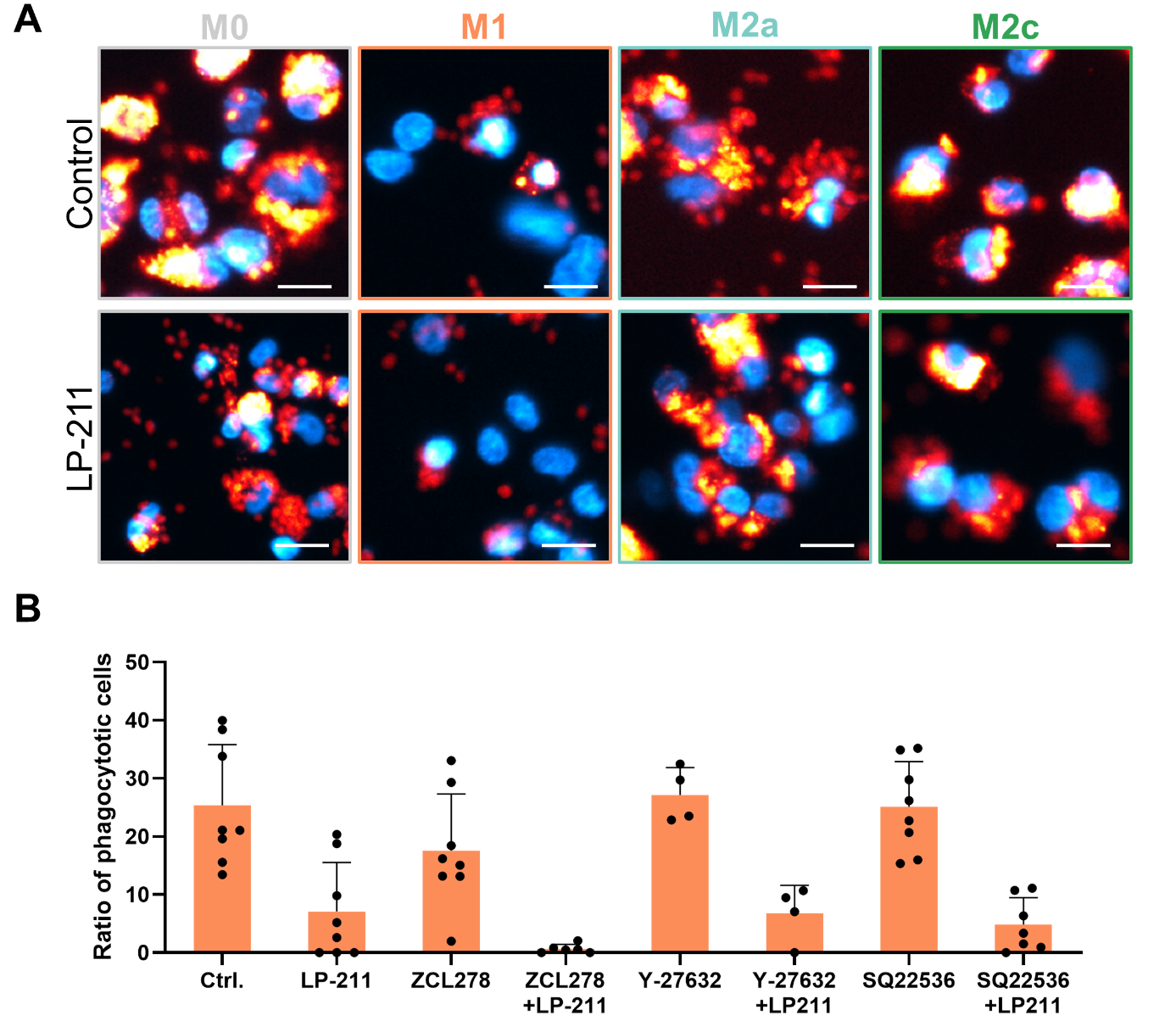


**Fig. S4 Modulation of phagocytosis upon inhibition of defined 5-HT7R signaling components**

**A:** Representative images of DAPI stained nuclei and Texas red-coupled Zymosan A particles in macrophages after control (upper panel) or LP-211 (lower panel) treatment at day 3 post differentiation. Scale bars 50 µm. **B:** Quantification of phagocytic ability of pro-inflammatory THP-1-derived M1‑like macrophages upon selective inhibition of 5-HT7R pathway components using LP-211 (10 µm, 48 h), ZCL278 (50 µm, 48 h), Y-27632 (10 µm, 48 h), SQ22536 (100 µm, 48 h) or control treatment (N≥4, each point displays the mean of 3 replicate wells).


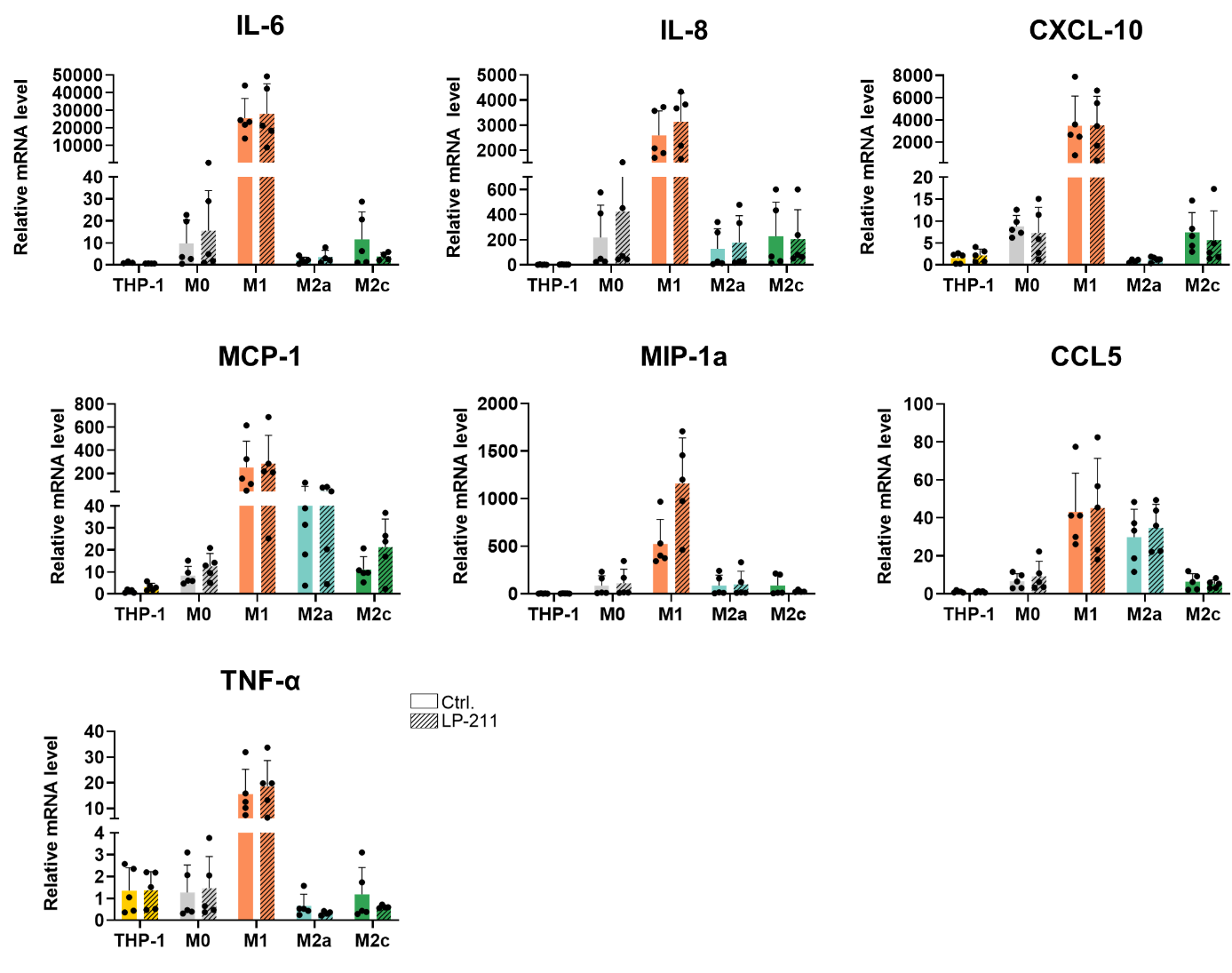


**Fig. S5 mRNA expression levels of selected cytokines and chemokines in THP-1-derived macrophages**

Quantification of mRNA levels of IL-6, IL-8, CXCL-10, MCP-1, MIP-1a, CCL5, TNF-α upon control and LP-211 (10 µm, 48 h) treatment (N=5). Statistical differences were analyzed using multiple t-tests (IL-6, MCP-1, CCL5) and multiple Mann-Whitney tests (IL-8, CXCL-10, MIP-1a, TNF-α) with Holm-Šídák method.


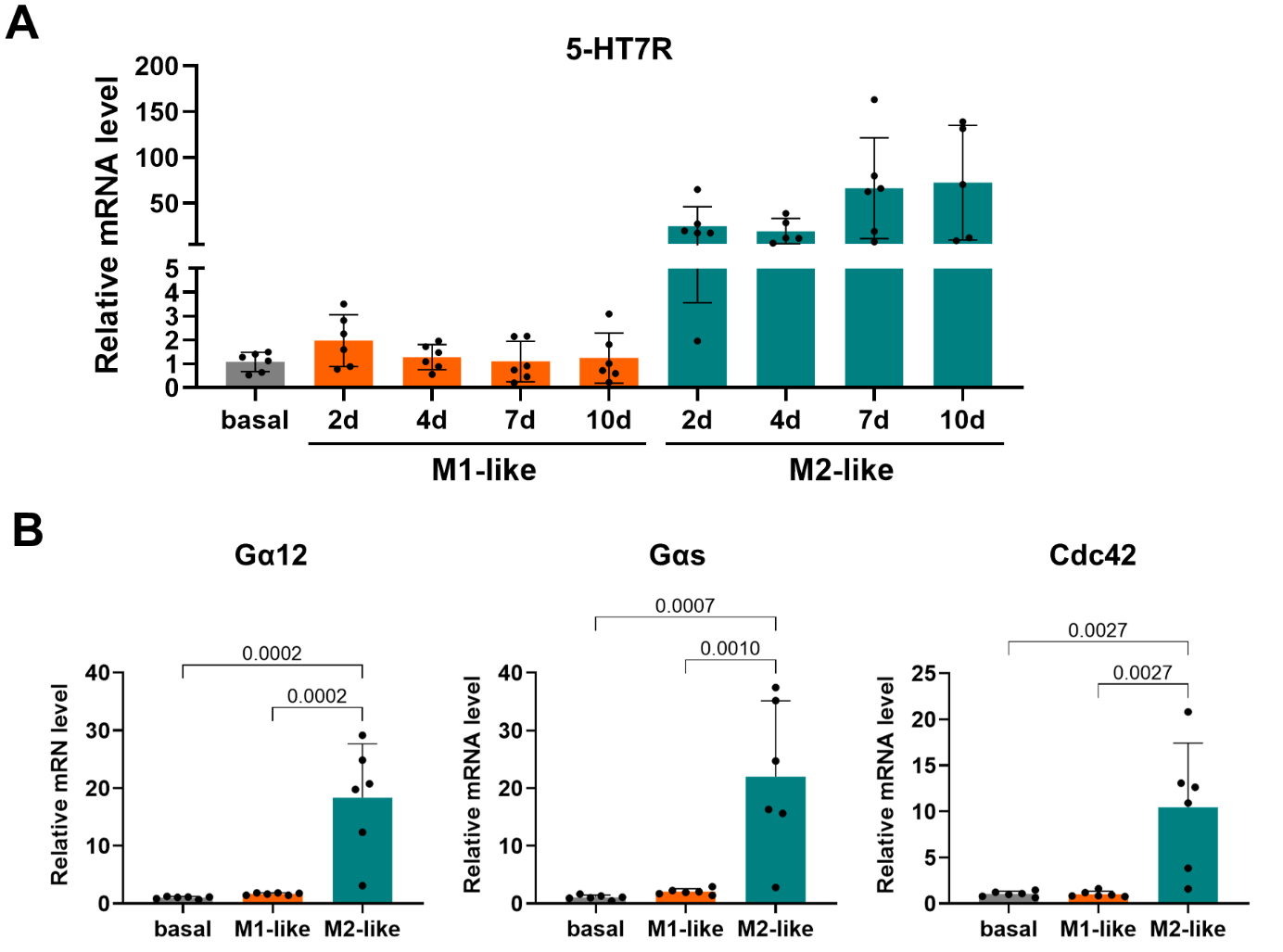


**Fig. S6 mRNA expression levels of 5-HT7R and its downstream effectors in CD14^+^ macrophages**

**A:** Time course of 5-HT7R mRNA expression during CD14^+^-derived macrophage polarization *in vitro*. **B:** mRNA expression levels of Gα12, Gαs and Cdc42 on day 7 of differentiation. Statistical differences were assessed using ordinary one-way ANOVA with Tukey’s multiple comparison test (N=6).
